# Inositol Polyphosphate Multikinase (*IPMK*), a Gene Coding for a Potential Moonlighting Protein, Contributes to Human Female Longevity

**DOI:** 10.3390/genes10020125

**Published:** 2019-02-08

**Authors:** Francesco De Rango, Paolina Crocco, Francesca Iannone, Adolfo Saiardi, Giuseppe Passarino, Serena Dato, Giuseppina Rose

**Affiliations:** 1 Department of Biology, Ecology and Earth Sciences, University of Calabria, 87036 Rende, Italy; francesco.derango@unical.it (F.D.R.); crocco.paola@gmail.com (P.C.); francescaiannonebio@gmail.com (F.I.); giuseppe.passarino@unical.it (G.P.); serena.dato@unical.it (S.D.); 2 MRC Laboratory for Molecular Cell Biology, University College London, London WC1E 6BT, UK; a.saiardi@ucl.ac.uk

**Keywords:** aging, longevity, survival, SNP, polymorphism, IPMK, inositol phosphates, gender-specific association, moonlighting protein

## Abstract

Biogerontological research highlighted a complex and dynamic connection between aging, health and longevity, partially determined by genetic factors. Multifunctional proteins with moonlighting features, by integrating different cellular activities in the space and time, may explain part of this complexity. Inositol Polyphosphate Multikinase (IPMK) is a potential moonlighting protein performing multiple unrelated functions. Initially identified as a key enzyme for inositol phosphates synthesis, small messengers regulating many aspects of cell physiology, IPMK is now implicated in a number of metabolic pathways affecting the aging process. IPMK regulates basic transcription, telomere homeostasis, nutrient-sensing, metabolism and oxidative stress. Here, we tested the hypothesis that the genetic variability of *IPMK* may affect human longevity. Single-SNP (single nuclear polymorphism), haplotype-based association tests as well as survival analysis pointed to the relevance of six out of fourteen genotyped SNPs for female longevity. In particular, haplotype analysis refined the association highlighting two SNPs, rs2790234 and rs6481383, as major contributing variants for longevity in women. Our work, the first to investigate the association between variants of *IPMK* and longevity, supports *IPMK* as a novel gender-specific genetic determinant of human longevity, playing a role in the complex network of genetic factors involved in human survival.

## 1. Introduction

In the last few decades, research on aging has seen progressive growth due to the social and medical burden correlated to the increase of the elderly population in developed countries. These efforts point towards a better understanding of the connections between aging, health, and longevity as they may provide useful insights for strategies to improve the wellbeing of the elderly. The results obtained in different research areas underscored the dynamic complexity of such connections [1]. These studies often identify genes involved in the regulation of the aging process that are also susceptibility loci of one or multiple age-related diseases. For instance, many genetic variants associated with increased disease risk are present with high frequency among the oldest individuals: this means that a disease “risk allele” can also be a pro-longevity variant [2,3]. In some cases, the same variant exhibits opposite effects on the development of different diseases, with potential differential impact on longevity [4,5]. Finally, genetic risk factors may change their impact on mortality risk during the life course, i.e., from detrimental in middle life to beneficial at advanced ages, very often in a gender-specific way [6,7]. This makes the identification of genes that robustly associate with longevity very challenging; in fact, despite the high number of studies aimed at highlighting the genetic contributors to long-life, only *APOE* and *FOXO3A* were consistently replicated in different populations [8].

Among the mechanisms that may account for this complexity are interactions between different genes (epistasis) or single nuclear polymorphism (SNP)–SNP interactions at gene level, gene–environment (internal and external) interactions and pleiotropic (including trade-off-like) effects of genes on different phenotypes. Alongside this, potential contributing factors could be genes coding for proteins with different, relevant and often unrelated functions, since they may integrate various cellular activities in space and time. This special category of multifunctional proteins defined as moonlighting proteins [9], does not include protein isoforms resulting from different RNA splice variants, gene fusions or proteins with pleiotropic effects [10]. A protein with potential moonlighting capability is the inositol polyphosphate multikinase (IPMK). 

This protein was initially discovered in budding yeast and named Arg82 for its ability to regulate arginine metabolism [11,12]. Mammalian IPMK has well-established roles in inositol phosphate metabolism as it converts inositol (1,4,5)-trisphosphate (IP_3_) to IP_4_ and IP_4_ to IP_5_ [13,14]. In addition to its kinase activity, IPMK can function as a nuclear phosphoinositide kinase (PI3-kinase), which produces PIP_3_ from PIP_2_ [15]. Through its PI3-kinase activity, IPMK activates Akt/PKB and its downstream signaling pathways [16]. In addition, it regulates several protein targets non-catalytically via protein–protein interactions, including cytosolic signaling factors such as the mammalian target of rapamycin complex 1 (mTORC1) [17] and the energy-sensing protein kinase AMPK [18]. Recently, Kim et al. [19] revealed that IPMK acts as an important regulator of Toll-like receptor (TLR)-induced innate immunity through its interaction with the tumor necrosis factor receptor–associated factor 6 (TRAF6). At the nuclear level, IPMK acts as a transcriptional coactivator for *p53* [20] and for serum response factor (SRF) signaling [21]. Finally, IPMK functions in the export of mRNA from the nucleus to the cytoplasm [22,23,24]. 

To our knowledge, there are no studies investigating the genetic variability of *IPMK* gene in human complex phenotypes; just one SNP, rs12570088, near to *IPMK* locus, was found related to the susceptibility to Alzheimer’s and Crohn diseases [25]. 

Given the compelling evidence demonstrating IPMK’s multifunctional nature and thus its classification as a moonlighting protein, the present paper addresses the hypothesis that *IPMK* genetic variability affects human aging and longevity. 

## 2. Materials and Methods 

### 2.1. Population Sample

We analysed five hundred sixty-eight unrelated subjects (252 men and 316 women aged 64–105 years), born in Calabria (South Italy) and recruited in the entire region through several campaigns, as previously reported [26]. Their Calabrian ancestry was ascertained up to the third generation. At baseline, all subjects were free of the major age-related pathologies (e.g., cancer, type-2 diabetes and cardiovascular diseases). The study was approved by the Ethical Committee of the University of Calabria (on 9-9-2004). Written informed consent was obtained from the subjects in accordance with institutional requirements and the Declaration of Helsinki principles.

The analyses were performed considering two sex- and age-specific groups obtained according to the survival functions of the Italian population from 1890 onward [27]. The two “thresholds of longevity” used to define these age classes were 88 years for men and 91 years for women. These cut-offs correspond to the point after which a significant negative change in the slope of the survival curve of the Italian population occurs. In particular, in the present study males younger than 88 and females younger than 91 years will be defined as controls (N = 309, mean age 74 years), while males older than 88 and females older than 91 years will be defined as cases (N = 259, mean age 96.9 years).

### 2.2. SNP Selection and Genotyping

We performed genotyping of 14 SNPs mapping within and nearby the *IPMK* gene, prioritized by a tagging approach. Analysis was performed by SEQUENOM MassArray iPLEX technology according to the procedure previously reported [28]. Sequenom Typer 4.0 Software was used for the management and analysis of the collected data. About 10% of the samples were reanalyzed and the concordance rate of the genotypes was higher than 99%.

### 2.3. Quality Control

After genotyping, samples were subjected to a battery of quality control (QC) tests. At sample level, subjects with a proportion of missing genotypes higher than 10% were dropped from the analysis. At SNP level, SNPs were excluded if they had a significant deviation from Hardy–Weinberg equilibrium (HWE, *p* < 0.05) in the control sample, a Missing Frequency (MiF) higher than 10% and a Minor Allele Frequency (MAF) lower than 5%. 

### 2.4. Functional Parameters 

#### 2.4.1. Disability

A modification of the Katz’ Index of activities of daily living (ADL) was used to assess the management of four everyday activities (toileting, getting up from bed, rising from a chair, walking around) [29]. For the analysis, ADL scores were dichotomized as 1 if the subject was able to perform every activity and 0 otherwise.

#### 2.4.2. Physical Performance

Evaluation of Hand Grip strength (HG) was performed through a handheld dynamometer (SMEDLEY’s dynamometer TTM, Tokyo, Japan) while the subject was sitting with the arm close to their body, by repeating the measure three times with the stronger hand. The maximum of these values was used in statistical analyses. When the test was not performed, it was indicated if it was because of physical disabilities or if the subject refused to participate.

#### 2.4.3. Cognitive Functioning

Screening of cognitive impairment was carried out by MMSE, a 30-point scale able to evaluate several different cognitive areas including memory, calculation, abstraction, judgment, visual–spatial ability and language [30]. MMSE scores range from 0 (lowest cognitive function) to 30 (highest cognitive function). MMSE scores were normalized for age and educational status, variables known to affect the result of the test. 

### 2.5. Statistical Methods

For each SNP, allele and genotype frequencies were estimated by gene counting from the observed genotypes. Hardy–Weinberg equilibrium (HWE) was tested by Fisher’s exact test. Pairwise measures of linkage disequilibrium (LD) between the analyzed loci was estimated by Haploview (https://www.broadinstitute.org/haploview/haploview). A logistic regression model was also used to evaluate the effect of genetic variability on the chance to reach very advanced age. Different genetic models (dominant, additive and recessive) were used to test association, using for each SNP the minor allele as reference. For each SNP the most likely genetic model was then estimated on the basis of minimum level of statistical significance (Wald test *p*-value).

As this study was exploratory, the *p*-values are reported without employing conservative statistical significance thresholding procedure (e.g., Bonferroni correction) as that could eliminate potentially important findings.

In order to evaluate if the detected effect of the polymorphisms on longevity may result in differential patterns of survival of the different relevant genotypes, we evaluated survival after 10 years from the baseline visit. Univariate survival analysis was carried out by the Kaplan–Meier method and survival curves compared by log-rank test. Subjects alive after the follow-up time were considered as censored, and this time was used as the censoring date in the survival analyses. In addition, hazard ratios (HR) and 95% confidence intervals (95% CI) were estimated by using Cox proportional hazard models taking into account age as a confounder variable.

Pairwise measures of linkage disequilibrium (LD) between the analyzed loci were calculated by Plink 1.9 [31] and plotted with the Haploview version 4.2 [32]. The amount of LD was quantified by Lewontin’s coefficient (D’). Haplotype-based association analysis within the generalized linear model (GLM) framework was used to model the effect of haplotypes on the probability to attain longevity, by the haplo.stats package of R. The haplo.score function of this package has been used to obtain the score statistics. Permutation-based *p*-values were used to evaluate the significance of the scores obtained (10,000 permutations). 

Statistical analyses have been performed using SNPassoc and surv packages of R [33].

## 3. Results

**Figure 1:**
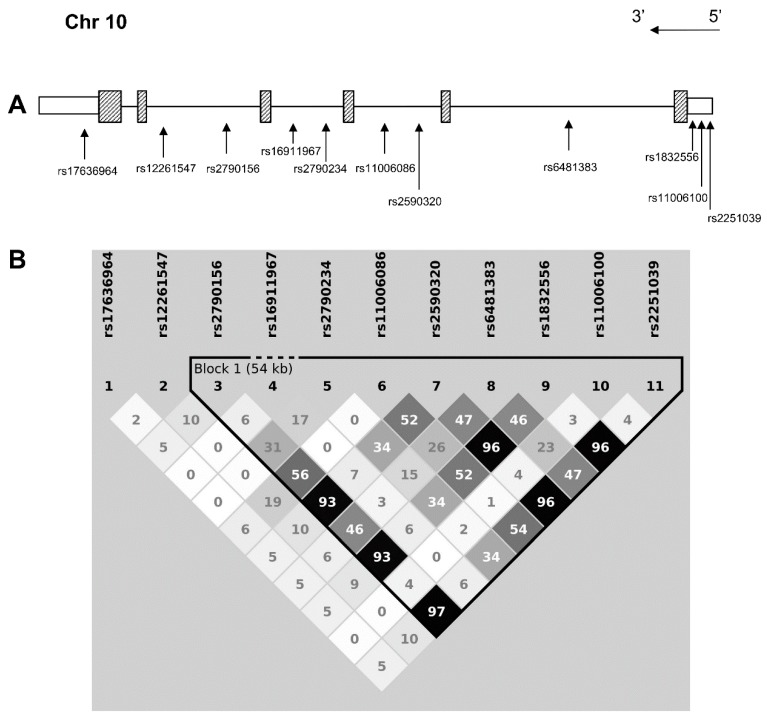
Schematic representation of (**A**) selected polymorphisms in the Inositol Polyphosphate Multikinase (*IPMK*) region; (**B**) linkage disequilibrium (r^2^ coefficient) among the single nuclear polymorphisms (SNPs).

Fourteen SNPs from about 76 kb genomic sequences spanning the *IPMK* gene were selected for examination by a tagging approach. The QC phase excluded three SNPs. In particular, two SNP were excluded due to MiF data higher than 10% (rs1698392, rs2440854) and one because it did not satisfy the HWE (rs2275443). Figure 1A shows the eleven high quality SNPs that were tested for association with longevity with their corresponding gene position, while panel B depicts the degree of LD between pairs of SNPs. 

### 3.1. Association with Longevity

#### 3.1.1. Single SNP Analysis

The general characteristics of the analyzed sample are described in Table 1. Since a number of studies highlighted gender- and age-specific associations with survival at advanced age, in this work we analyzed the role of *IPMK* SNPs in the predisposition to become long-lived in gender subgroups. 

From the results of the logistic regression analysis shown in Table 2, it can be seen that significant differences between the long-lived subjects and younger controls are present among females only. In particular, six out of eleven markers (in order: rs2790156-G/A, rs2790234-C/G, rs2590320-C/A, rs6481383-C/T, rs1832556-G/A, rs2251039-C/T) were significantly associated with the longevity phenotype under a dominant model of inheritance. For all the SNPs, the presence of the minor allele conferred decreased odds to reach advanced old age. rs2790234 showed the greatest impact (Odd Ratio, OR, 0.33; 95% Confidence Interval, CI, 0.16–0.67; P = 0.00225), while association of similar magnitude was observed for the other five polymorphisms with ORs (95% CI) of 0.62, 0.572, 0.592, 0.592 and 0.612 (all *p*-values < 0.05) for rs2790156, rs2590320, rs6481383, rs1832556, rs2251039, respectively). 

#### 3.1.2. Haplotype-Based Analysis

To further explore the association of the entire region with longevity, we performed a haplotype analysis among the six SNPs associated with longevity. As shown in Figure 1B, all six SNPs lie in a large LD block, with rs2790156, rs2590320, rs1832556 and rs2251039 in strong LD and rs2790234 and rs6481383 in a weak linkage. Among all possible haplotypes, we found only four combinations: G-C-C-C-G-C, 61%; G-C-C-T-G-C, 16%; A-C-A-T-A-T, 14%; A-G-A-T-A-T, 7% (Table 3). In line with the single locus analysis, we found a negative association of the minor allele combination A-G-A-T-A-T with longevity in females (*p*-value = 0.002). On the contrary, a positive association was observed for the opposite combination G-C-C-C-G-C (*p*-value = 0.024). A deep analysis of the associated haplotypes showed that the strength of these associations was influenced by the allelic status at rs2790234 and rs6481383. Indeed, while A-**G**-A-T-A-T is significantly associated, the A-**C**-A-T-A-T is not; likewise, while G-C-C-**C**-G-C showed an effect on longevity, this was not true for G-C-C-**T**-G-C.

### 3.2. Association with Survival 

**Figure 2:**
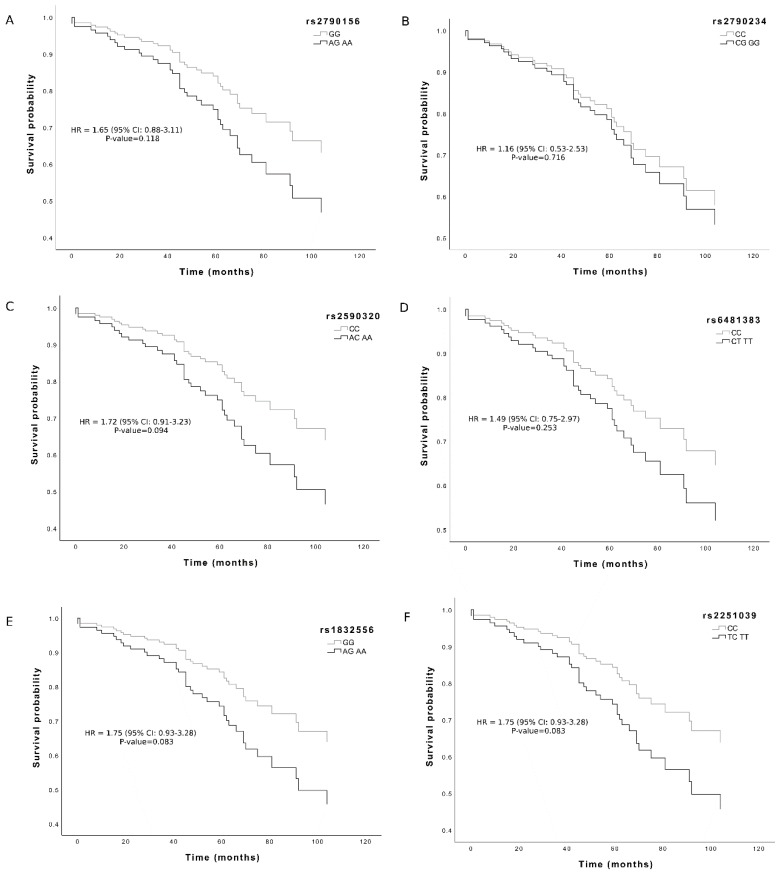
Survival functions of female carriers of minor allele (black) vs non-carriers (grey) of IPMK variants. (**A**) rs2790156; (**B**) rs2790234; (**C**) rs2590320; (**D**) rs6481383; (**E**) rs1832556; (**F**) rs2251039. Time is expressed in months, where 0 is considered the time of recruitment and each individual is followed up for survival status till death. The Cox regression was adjusted for age. Hazard ratio (HR) value, confidence interval and *p*-value from Cox regression analysis are reported inside the figure.

By using 10 years of follow-up survival data, we investigated if the single variants associated with female longevity also influenced the survival of the younger cohort. As shown in Figure 2, consistent with the detrimental effect on longevity, we found a trend toward significance for three out of six variants associated with longevity, rs2590320, rs1832556 and rs2251039, with HR values 1.72 (0.91–3.23), 1.75 (0.93–3.28), 1.75 (0.93–3.28) respectively (*p* < 0.1). we could not perform a haplotype-based survival analysis because a classification of carriers or non- carriers would reduce the size of the two classes too much.

### 3.3. Association with Functional Parameters

To investigate whether the variants in *IPMK* gene also concur to determine the age-related physiological decline, we analyzed the SNPs in relation to markers of physical (ADL and Hand Grip) and cognitive (MMSE) performance. No significant association was detected (data not shown). As a result, we conclude that *IPMK* has an effect on survival and longevity independently of the tested variables.

## 4. Discussion

Our study was designed to test the hypothesis that genetic variability at the *IPMK* locus contributes to survival to very old age. We provided evidence that polymorphisms in this gene significantly affect the females’ chance of survival to old age, a result that implicates IPMK, a multifunctional protein with potential moonlighting functions, as a significant contributor to gender differences in longevity. 

The gender difference in life expectancy and mortality, including survival to extreme age, as well as prevalence and incidence of the most important age-related diseases, is supported by a huge amount of clinical and demographic data [1,34]. In almost all modern populations, females live longer than males and this has been attributed to a particular combination of genetic factors, environmental factors (nutrition and stress), sex hormones and immunity, along with socio-economic and cultural factors [35,36]. Gender-specific longevity alleles were identified for a long time [37,38] and recently confirmed by genome-wide association studies (GWAs) [39,40]. These studies also indicated that different pathways contribute to longevity in men and women; for instance, paths involved in inflammation and immunity emerged as male-specific, while those involved in PGC-1α (PPARγ coactivator-1α) function and tryptophan metabolism emerged as female specific [40]. It is intriguing that many members of these pathways are known to perform diverse unrelated functions, behaving as moonlighting proteins [41,42]. As previously discussed, proteins with moonlighting properties may, in part, explain the complex role of genetic factors in determining the longevity phenotype, including gender-linked effects. In this sense, IPMK is surely a possible player because of multifunctional protein feature. IPMK catalyzes key steps leading to the synthesis of inositol pyrophosphates [43]. These molecules, that as the name indicates contain one or more pyrophosphate moiety, control several aspects of cell physiology essential for cell survival. Inositol pyrophosphate regulate telomere length [44], vesicular trafficking [45], DNA recombination [46], ROS signaling [47] and energetic metabolism [48], likely by controlling cellular phosphate homeostasis [49,50]. In fact, these molecules regulate the pathophysiology of metabolic disorders such diabetes and obesity [51,52]. The numerous and distinct connections between metabolism and aging that research is highlighting [53,54] suggest that inositol pyrophosphate might be relevant to the aging process. Remarkably, the knockout of inositol hexakisphosphate kinase 3 (IP6K3), an essential enzyme for inositol pyrophosphates synthesis, results in altered metabolism and extends mice lifespan [55]. Furthermore, we associated two SNPs in the 5′-flanking promoter region of the *IP6K3* gene with the susceptibility to late onset Alzheimer’s disease (LOAD) [28]. Therefore, the catalytic activity of IPMK, indispensable for inositol pyrophosphate synthesis, could be ultimately important for controlling metabolism and lifespan. Independently from its enzymatic activity, through protein–protein interaction, IPMK acts as a signaling hub in regulating nutrient and energetic pathways, including mTOR and AMPK [17,56]. The inhibition of mTORC1 pathway components extends lifespan and confers protection against an increasing list of age-related diseases, while on the contrary its over-induction leads to a higher risk of age-related diseases and decreased lifespan [57,58] and references therein]. A role in the control of cell survival and death has been also highlighted. A small deletion that leads a truncated form of IMPK was found to reduce the activation of *p53* and increase the resistance to apoptosis of cancer cell lines [59]. Moreover, Davey et al. found that IPMK plays an important role in necroptosis, a form of regulated cell death prompted by injury and infection [60]. Both apoptosis and necroptosis impact on a variety of processes governing cell physiology and homeostasis with implications in health and disease [61].

It is interesting to note that many of the processes in which IPMK participates influence aging in a gender specific manner. For instance, some authors have suggested that gender differences in lifespan are due to gender-specific susceptibility to oxidative stress [62] and yet gender-specific survival is associated with sex differences in telomere dynamics [63]. It has also been demonstrated that rapamycin, the inhibitor of mTOR signaling, extends lifespan in dose- and gender-specific ways [64] and that there are sex-differences in muscle AMPK activation [65]. 

On the whole, these studies corroborate our findings that six *IPMK* SNPs significantly affect female longevity. Haplotype analysis confirmed the single SNP analyses, identifying an advantageous effect in carriers of the haplotype G-C-C-C-G-C; conversely the A-G-A-T-A-T haplotype is a disadvantageous combination for longevity. Haplotypes analysis allowed us to establish that among the six SNPs, two SNPs, rs2790234-C/G and rs6481383-C/T, were more likely to have an effect on lifespan, the minor rs2790234-G allele conferring a longevity disadvantage and the major rs6481383-C allele having a beneficial effect on the trait. The absence of correlation with survival and parameters of quality of aging suggests that these SNPs play a major role on the probability to achieve longevity.

Since we have no demonstrable functional explanation for the association as both SNPs occur in large intronic regions, it is difficult to evaluate their significance at this time. Moreover, both SNPs lie in a large region of LD, which likely contain hundreds of polymorphisms with one or more others that may have a direct contribution. Thus, our findings could be relevant for future investigations.

This study has some limitations that merit consideration. First, the sample size is rather small, which limits its statistical power. The sample size might have influenced the significance of survival analysis, so only trends have been identified. However, this result may also depend on the follow-up time of 10 years, not sufficient to draw long-term conclusions on the effect of genetic variants with a minor effect on survival. Therefore, the sample size should be increased and further explorations in additional populations and other countries are needed before drawing further conclusions. Another possible drawback is the lack of a proper correction for multiple testing. However, it should be noted that this was a pilot study, the first to analyze the association of *IPMK* variability in human aging and longevity, so a Bonferroni correction would have eliminated potentially important findings if applied. Furthermore, because associated SNPs are intronic, experimental evidence in support of the hypothesis that the detrimental genotypes influence the function of the protein should be carried out. 

## 5. Conclusions

Specific *IPMK* haplotypes affect lifespan in women. Although our studies do not allow definitive conclusions, we believe that our findings can provide a basis for future studies to better clarify the basic mechanisms linking *IPMK* to female longevity and potential targets for realizing gender-specific therapeutic interventions. Finally, we believe that proteins with moonlighting capabilities, such as IPMK, could represent one of the factors that makes it difficult to disentangle the genetic complexity of the longevity phenotype.

## Figures and Tables

**Table 1:** General characteristics and post-survey mortality in the analyzed sample.

	Elderly Subjects	Long-Lived Subjects
N (age)	309 (74.06 ± 6.95)	259 (96.92 ± 3.72)
Females %	49.5%	63.0%
Height (cm)	160.6 (9.7)	151.4 (9.5)
BMI	26.9 (4.2)	23.21 (4.1)
HG strength [Kg (SD)]	21.89 (9.9)	13.04 (6.4)
ADL* (% Disabled)	17%	69%
MMSE	23.3 (5.4)	14.0 (6.8)

ADL, Activity Daily Living; HG, Hand Grip; BMI, Body Mass Index; MMSE, Mini Mental State Examination; for each parameter, mean value and standard deviation, in brackets, are shown. *Participants were defined as “not disabled” if independent in all items and “disabled” if dependent in at least one item.

**Table 2:** Results of the logistic regression models for *IPMK* SNPs in the sample divided by sex.

**(a)** **Females**			
SNP (Major/Minor Allele)	OR	95% CI	*p*-value
rs17636964 (G/C)	1.48	0.82–2.662	0.185
rs12261547 (G/C)	1.39	0.62–3.11	0.415
rs2790156 (G/A)	0.61	0.38–0.98	0.042
rs16911967 (G/C)	0.40	0.12–1.33	0.136
rs2790234 (C/G)	0.33	0.16–0.67	0.002
rs11006086 (T/C)	0.67	0.34–1.32	0.255
rs2590320 (C/A)	0.57	0.36–0.91	0.019
rs6481383 (C/T)	0.59	0.37–0.94	0.026
rs1832556 (G/A)	0.59	0.37–0.94	0.028
rs11006100 (T/A)	0.69	0.42–1.14	0.154
rs2251039 (C/T)	0.61	0.38–0.97	0.038
**(b)** **Males**			
SNP	OR	95% CI	*p*-value
rs17636964 (G/C)	0.98	0.50–1.94	0.974
rs12261547 (G/C)	0.52	0.16–1.66	0.272
rs2790156 (G/A)	0.79	0.46–1.35	0.397
rs16911967 (G/C)	2.08	0.54–7.96	0.283
rs2790234 (C/G)	0.98	0.49–1.94	0.959
rs11006086 (T/C)	0.76	0.31–1.85	0.550
rs2590320 (C/A)	0.81	0.48–1.37	0.436
rs6481383 (C/T)	1.10	0.65–1.87	0.713
rs1832556 (G/A)	0.81	0.47–1.37	0.436
rs11006100 (T/A)	1.73	0.98–3.04	0.056
rs2251039 (C/T)	0.78	0.46–1.34	0.377

OR: Odd Ratio; CI: Confidence Interval.

**Table 3:** Estimation of haplotype frequencies in the IPMK SNPs (in order: rs2790156, rs2790234, rs2590320, rs6481383, rs1832556, rs2251039) and association with longevity in the female sample.

Haplotype	Frequency	Score	*p*-Value *
A-G-A-T-A-T	0.067	−2.897	0.002
A-C-A-T-A-T	0.138	−0.668	0.483
G-C-C-T-G-C	0.161	−0.353	0.715
G-C-C-C-G-C	0.616	2.155	0.024

* simulated *p*-value obtained by Monte Carlo replication up to 10,000 bootstraps.
